# The Ethics of Clinical Ethics

**DOI:** 10.1007/s10730-024-09544-3

**Published:** 2024-11-29

**Authors:** Matthew Shea

**Affiliations:** Philosophy Department, Franciscan University of Steubenville, Steubenville, OH USA

**Keywords:** Clinical ethics, Ethics consultation, Metaethics, ASBH

## Abstract

The concept *ethics* defines health care ethics as a professional practice. Yet the meaning of “ethics” is often unclear in the theory and practice of clinical ethics. Clarity on this matter is crucial for understanding the nature of clinical ethics and for debates about the professional identity and proper role of ethicists, the sort of training and skills they should possess, and whether they have ethics expertise. This article examines two different ways the ethics of clinical ethics can be understood: Real Ethics, which consists of objective moral norms grounded in moral truth; and Conventional Ethics, which consists of conventional norms grounded in bioethical consensus. Drawing on the bioethics literature and features of professional practice, it shows that Conventional Ethics is the dominant paradigm. Then it presents a critique of Conventional Ethics, arguing that it cannot avoid the challenge of moral pluralism, it fails to address vitally important moral questions, and it is incapable of providing an essential service to the people ethicists aim to help. It ends with suggestions about how the practice of clinical ethics might overcome these problems.

## Introduction

In her famous article “Modern Moral Philosophy,” G.E.M. Anscombe highlights the discrepancy between ancient and modern conceptions of ethics, writing: “If someone professes to be expounding Aristotle and talks in a modern fashion about ‘moral’ such-and-such, he must be very imperceptive if he does not constantly feel like someone whose jaws have somehow got out of alignment: the teeth don’t come together in a proper bite” (1958, p. 2). A similar misalignment can happen when clinical ethicists talk about what is “ethical.”

The concept *ethics* defines the nature of health care ethics as a professional practice. When engaging in case consultation, education, and policymaking, clinical ethicists constantly refer to what is “ethically” appropriate, justified, permissible, impermissible, obligatory, etc.^1^ For such statements to be clear and meaningful, we must know what “ethics” picks out. And yet, surprisingly, the concept *ethics* and the term “ethics” are often ambiguous. As H. Tristram Engelhardt, Jr. observes, despite the large academic literature on clinical ethics and the growing movement to professionalize the practice, “there remains the central challenge of giving an adequate account of the nature of the ethics of clinical ethics consultation” (2011, p. 134). It is often unclear how the notion of ethics operative in clinical ethics is related to other ones, such as those found in moral philosophy. The meaning of ethics is closely linked to current debates about the professional identity and proper role of ethicists, the sort of training and skills they should possess, and whether they have ethics expertise. For all of these reasons, the ethics of clinical ethics deserves careful analysis.

This article explores the meaning of ethics in secular clinical health care ethics.^2^ First, I lay out two different ways that ethics can be understood, which I call Real Ethics and Conventional Ethics. Drawing on canonical sources in the literature, opinions of prominent bioethicists, and features of professional praxis, I show that the second view is the dominant one in clinical ethics. Next, I raise a series of problems for the Conventional Ethics approach, arguing that it cannot avoid the challenge of moral pluralism, it fails to address vitally important moral questions, and it is incapable of providing an essential service to the people ethicists aim to help. I end with some suggestions about how the practice of clinical ethics could overcome these problems.

## Two Candidates for the Ethics of Clinical Ethics

This section contains a descriptive metaethical analysis of the meaning of ethics in clinical ethics, in both the scholarly literature and real-life practice. We can start with the ordinary notion of ethics. In a basic and commonplace sense, ethics deals with how we ought to live, be, and act; with concepts such as good and bad, virtue and vice, and right and wrong. Ethics is a *normative* subject: it does not just describe what *is* but evaluates and prescribes what *ought* to be. Ethics is about *morality* and moral normativity rather than other forms of normativity, such as law, epistemology, and etiquette (Pojman, 2016, ch. 1). The ordinary notion of ethics is reflected in standard dictionary definitions of the term: “the discipline dealing with what is good and bad and with moral duty and obligation” (*Merriam-Webster Dictionary*); “the study of what is morally right and wrong, or a set of beliefs about what is morally right and wrong” (*Cambridge Academic Content Dictionary*); “The branch of knowledge or study dealing with moral principles” (*Oxford English Dictionary*).

The ordinary notion of ethics aligns with the first candidate for the ethics of clinical ethics: *Real Ethics*. It can be defined as the attempt to know and to do what is morally good, right, and virtuous in a fundamental, ultimate, and unqualified sense. Some of the core issues it addresses are the nature of the good, the right, and virtue; the moral evaluation of action and character; systems of moral principles, rules, obligations, rights, and virtues; and norms of practical rationality. Like academic moral philosophy, it is concerned with *moral* norms rather than other kinds of norms, such as legal, political, and social norms. Although there can be overlap between them, morality is independent of and irreducible to law, politics, social convention, and so on.^3^ Unlike academic moral philosophy, Real Ethics is essentially practical. It involves both a theoretical component (inquiring and knowing) and a practical component (doing and being). Real Ethics aims at moral truth, and the goal is to arrive at correct moral beliefs, choices, and actions. In these ways, it is committed to de facto *ethical objectivism*: the standard of ethical justification is objective and universal; the validity of ethical norms is independent of the beliefs, attitudes, and practices of human individuals and groups (Pojman, 2016, ch. 3).^4^

On an understanding of clinical ethics as Real Ethics, when ethicists perform ethical analysis, give recommendations, and engage in conflict resolution in active patient cases, the goal is to determine what is morally good/right/virtuous and see that it is done. Because it is concerned with moral truth, Real Ethics is mostly substantive as opposed to procedural. The primary focus is not the proper decision maker or process—who should make the decision and how it should be made—but the morally appropriate course of action—what the decision ought to be.^5^

Endorsements of the Real Ethics paradigm can be found in some canonical works in the bioethics literature. For example, the opening chapter of *Fletcher’s Introduction to Clinical Ethics* says, “Ethics may be defined as the analysis, study, or consideration of morality; here morality refers to what is considered ‘good’ or ‘right’” (Fletcher et al., 2005, p. 3). Bernard Lo’s *Resolving Ethical Dilemmas* provides a more detailed definition of ethics:

We use the term *ethics* to refer to judgments about what is right or wrong and worthy of praise or blame. This refers to moral judgments about right and wrong, not biotechnical or clinical judgments about the most effective or safest test or treatment….

Ethics also refers to a branch of philosophy that deals with the “principles governing ideal human character.” To philosophers, ethics focuses on the reasons *why* an action is considered right or wrong. It asks people to justify their positions and beliefs by rational arguments that can persuade others who may not share their specific cultural or religious affiliation. (2020, pp. 3, 6)

Tom Beauchamp and James Childress’s *Principles of Biomedical Ethics* also seems to operate with an understanding of ethics as Real Ethics:

*General normative ethics* addresses the question, “Which general moral norms should we use to guide and evaluate conduct, and why?” Ethical theories seek to identify and justify these norms, which are often referred to as principles, rules, rights, or virtues….

The term *practical ethics*, as used here, is synonymous with *applied ethics* and stands in contrast to *theoretical ethics*. *Practical ethics* refers to the use of moral concepts and norms in deliberations about moral problems, practices, and policies in professions, institutions, and public policy. (2019, pp. 1–2)

Even if these interpretations turn out to be incorrect, the Real Ethics model is adopted by some scholars and practitioners of clinical ethics.^6^  

Although Real Ethics aligns with the ordinary notion of ethics and is endorsed by prominent bioethicists, there are good reasons to think that the ethics of clinical ethics is not Real Ethics. Consider the following propositions:

1. Patients have an ethical right to refuse any and all treatment, no matter the reason, provided they have decision-making capacity and their refusal does not put others at risk of harm.
2. Surrogate decision makers are ethically required to make decisions on the basis of the patient’s known wishes, or, if these are unknown or inapplicable, on the basis of substituted judgment, or, if neither of these can be used, on the basis of the patient’s best interests.
3. There is no ethically relevant difference between withholding and withdrawing treatment. If it is ethically permissible to withhold a particular treatment, then it is ethically permissible to withdraw the same treatment, all else being equal.
4. It is ethically impermissible to administer pain medication with the intention of bringing about the patient’s death; although it can be permissible to administer pain medication with the intention of relieving the patient’s suffering even when doing so will hasten death, provided the patient’s death is unintended and there is a proportionate reason to do so.
5. If a patient is declared dead according to neurological criteria, it is ethically permissible to procure the patient’s organs with the consent of the patient or surrogate.
6. Artificial nutrition and hydration are not ethically different from other kinds of life-sustaining treatment, and they justifiably can be withheld or withdrawn for the same reasons.
7. Parents do not have an ethical right to refuse recommended life-saving treatment for pediatric patients on the basis of their own religious beliefs.
8. If a treatment is medically futile or inappropriate, physicians are not ethically obligated to provide it and are justified in refusing to do so, even if the patient or family requests it.
9. Health care providers have an ethical right to exercise conscientious objection and refuse to perform interventions they believe are immoral.
10. It is ethically permissible for physicians to perform elective, early-term abortions for patients who have made an informed, autonomous request for the procedure.

According to the prevailing consensus in secular bioethics, all of these statements are examples of valid, authoritative, and established ethical norms that enjoy widespread endorsement and practical application (cf. Brummett, 2021; Brummett and Eberl, 2022).

None of them, however, is endorsed by all persons from all moral and religious perspectives. To take three obvious examples, the moral issues surrounding medical futility, conscientious objection, and abortion are hotly disputed, with rival parties claiming to have moral truth on their side. The same can be said, with varying levels of disagreement, for a host of other moral issues in health care: the absolute right to refuse treatment; the ethical standards for informed consent and surrogate decision making; the moral equivalence of withholding and withdrawing treatment; the moral significance of intention; the definition of death and the dead donor rule; the obligation to provide artificial nutrition and hydration; the right of parents to refuse medical treatment for their children; the just distribution of medical resources; the moral status of some human beings (e.g., the unborn and permanently unconscious); and the morality of euthanasia and physician-assisted suicide, gender-related treatments, genetic engineering, and so on. As many thinkers have demonstrated, there is widespread moral pluralism and deep moral disagreement in our society, which manifests in conflicting moral beliefs and values in the health care setting (Engelhardt, 1996; MacIntyre, 2007). Both the highly controversial issues and many of the mundane rules in health care ethics are subjects of variation and dispute across moral and religious traditions, which have conflicting views of what is morally correct. Indeed, the challenge of moral pluralism is the central objection to the Real Ethics paradigm of clinical ethics (Engelhardt, 1996).

Now, depending on one’s moral views, one might think that some or all of the ten ethical statements listed above express moral truths. But it is widely held that in the context of clinical ethics, statements like (1)–(10) are not meant to be understood as moral truths but as a different kind of claim. When an ethics consultant makes a statement like (10) and advises that abortion is ethically permissible in a particular case, the consultant is not making a statement about the morality of abortion in a fundamental, ultimate, and unqualified sense, i.e., the way it would be addressed by Real Ethics. When ethical conflict and uncertainty is addressed in the clinical setting, it is not guided by moral truth. That is to say, the ethical norms themselves and the recommendations of ethicists are not supposed to track moral truth or help others arrive at the morally correct course of action.

David Adams, reflecting on the question of whether ethicists have ethics expertise, asks:

What sorts of answers to moral questions might we expect an ethics expert to provide? Here is one possibility: The specialist we are imagining is someone who gives *correct* answers. She is a *moral* expert, where this means an individual who can reliably arrive at correct answers to a variety of questions in practical normative ethics or who makes practical moral judgments that are dependably morally true—or at least someone who can do these things with a frequency exceeding that of the non-expert….

[W]ith some possible exceptions, almost no one in the literature defends the claim that [clinical ethicists] possess *moral* expertise in this sense—indeed, the attribution of such expertise to hospital ethicists is explicitly rejected in several sources. The [ethics consultant] is not someone who claims to deliver *right* or *true* answers to the puzzled and conflicted. (2018, p. 212)

The practice of clinical ethics is not an open inquiry into moral truth with all possible answers on the table, with the goal of making recommendations that correspond to moral truth and ensuring that goods are promoted, actions are right, and persons are virtuous. Paradoxically, the goal of clinical ethics is not to figure out what is morally right and see that the right thing is done, contrary to what one might suppose about a field with “ethics” in the name. (This is one of the reasons why ethicists might feel like their “teeth don’t come together in a proper bite” when talking about ethics, as Anscombe would put it.) Almost all the time, the ethical question, principle, or debate is already settled by the established ethical norms, like the ten listed above, and the ethicist is professionally bound to make recommendations in accordance with them.^7^ Therefore, the understanding of ethics at play cannot be Real Ethics, which does aim at moral truth and morally correct action.

This brings us to the second candidate for the ethics of clinical ethics: *Conventional Ethics*. It can be defined as the attempt to know and to do what is ethically appropriate in a conventional (rather than moral) sense. According to this approach, statements like (1)–(10) are conventional norms operative in professional practice, law, and institutional policy, which are meant to reflect bioethical consensus, i.e., the consensus among bioethicists, health care providers, and society, as reflected in the bioethics literature, professional society statements, law, and public policy (Turner et al., 2024). The norms in question are lumped together under the heading of “ethics,” which is due more to positivistic stipulation than to anything about the norms themselves that makes them inherently ethical. On this view, the ethics of clinical ethics tracks *convention*—“established,” “prevailing,” “received,” or “settled” ethical standards—rather than morality or objective moral truth. Although conventional norms can *coincide* with moral truths (e.g., according to both convention and morality, it is wrong for physicians to refuse to treat patients based on their race), whatever connection might exist between them is only *contingent*, *accidental*, and *extrinsic* to clinical ethics itself.^8^ Conventional norms are not universal but particular to a time, place, and cultural context. In these ways, Conventional Ethics is committed to de facto *ethical relativism*: the standard of ethical justification is relative; the validity of ethical norms is relative to and dependent on human beliefs, attitudes, or practices.^9^ More specifically, it is a version of conventional relativism—the ethical standard is a group’s socially constructed code—rather than subjectivism—the ethical standard is an individual’s code—or objectivism—the ethical standard is a universal moral law (Pojman, 2016, ch. 2).^10^

The main advantage of Conventional Ethics is supposed to be that, unlike Real Ethics, it can avoid the problem of moral pluralism because it does not depend on substantive, “content-full” moral systems or commitments. Its goal is not moral truth, and it is primarily procedural in nature, focusing on the appropriate decision maker as opposed to the morally correct decision. Because it allegedly bypasses the intractable moral disputes found in both academic bioethics and the clinical setting, Conventional Ethics is considered more viable than Real Ethics.

The Conventional Ethics paradigm is the most consistent with the theory and practice of clinical ethics in the secular American context. It is the majority view in the literature and the one that fits best with the real-life practice of most clinical ethicists. The remainder of this section analyzes the Conventional Ethics approach in more detail and provides evidence for the claim that it is the mainstream understanding of ethics.

First, take the *Core Competencies for Health Care Ethics Consultation* put forward by the American Society for Bioethics and Humanities (ASBH, 2011). This document is the best indicator of the dominant view of clinical ethics, because ASBH is the largest and most influential professional bioethics organization in the country and the only one that tries to provide authoritative standards for clinical ethics consultation. *Core Competencies* does not explicitly address the nature of ethics—which is surprising for a document about ethics consultation—but it contains clear evidence for the Conventional Ethics interpretation. ASBH equates “ethical” with “value-laden” and understands ethical issues as those involving uncertainty or conflict about values (2011, p. 4).^11^ According to ASBH, “The general goal of [ethics consultation] is to improve the quality of health care through the identification, analysis, and resolution of ethical questions or concerns” (2011, p. 3) Additional goals are to “identify and analyze the nature of the value uncertainty or conflict,” “facilitate resolution of conflicts,” and “promote practices consistent with ethical norms and standards,” all with an eye toward “building an ethically supportable consensus” (2011, pp. 3, 6).

In the part of *Core Competencies* on evaluating the quality of ethics consultation services, there is a section devoted to consultation outcomes, one of which is “ethicality.” This is a vague and uncommon term, which the *Oxford English Dictionary* defines as “The quality or state of being ethical (in various senses).” For ASBH, ethicality is understood as *conformity to established ethical standards*: “To evaluate ethicality as the outcome of an ethics consultation, one would have to determine whether ethics consultation resulted in decisions or actions that are consistent with established ethical standards” (2011, p. 39). The example offered to illustrate the notion of ethicality reinforces that ASBH is not referring to objective moral truths, but to social conventions that can change over time: “consider a case in which there is a question about whether a patient should be informed about a medical error that occurred in his or her care that resulted in serious harm. The *(relatively newly established)* ethical standard is that such patients should be informed” (2011, p. 39, emphasis added).

ASBH identifies three sets of skills required for ethics consultation: ethical analysis skills, process skills, and interpersonal skills. One of the skills listed under ethical analysis is the ability to “access relevant ethics knowledge (e.g., healthcare ethics law, institutional policy, professional codes, research/scholarship, and religious teachings)” (2011, p. 22). Contrary to the traditional definition of knowledge as justified true belief, nothing in this conception of ethics knowledge requires any connection to truth, and it is questionable whether it requires epistemic justification either. Moreover, it is implausible to think that this sort of ethics knowledge confers actual *moral justification* on the ethicist’s judgments and recommendations. As Adams explains,

The consultant must be competent to cull from literature, policy, and practice information about what options are allowed and report out those finding to the parties involved in conflict or uncertainty. To call this “ethics knowledge” is, however, contentious. Ethics fundamentally concerns critical reflection upon, and justification of, moral beliefs and judgments. What [proponents of Conventional Ethics] (and American Society for Bioethics and Humanities) delineate has more to do with *describing* what is or isn’t allowed than about *justifying* moral claims; the ethics consultant is more an expert about what is *said* to be moral than an expert in reasoning about what is moral.” (2013, p. 28)

The content of ethics knowledge, on this construal, is descriptive rather than normative.

*Core Competencies* lays out three different models of ethics consultation: “authoritarian,” “pure consensus,” and “ethics facilitation.” The discussion of these models contains illuminating clues about how ASBH understands ethics. The pure consensus approach, which aims at mere agreement among parties, is rejected because the consensus could “fall outside the boundaries of widely accepted ethical and legal norms and standards,” and this model “fails to incorporate the importance of ethically justified norms or values” (2011, p. 6). By contrast, the ethics facilitation approach, which ASBH favors, stays “within the bounds of ethical and legal standards” and aims to facilitate consensus within the range of ethically acceptable options, i.e., “options that are all ethically justifiable and consistent with prevailing ethical and legal standards” (2011, p. 9). According to ASBH, ethicists’ recommendations “should comport with the bioethics literature, medical literature, other relevant scholarly literature, current professional and practice standards in the field of [ethics consultation], statutes, judicial opinions, and pertinent institutional policies” (2011, p. 6). These sources taken together appear to serve as the normative standard for clinical ethics. In a revealing passage, the document asserts that “the ‘right’ substantive decision is ultimately the responsibility of the ethically appropriate decision maker(s) (generally the patient, the surrogate, the healthcare professional, the institution, or, at times, some combination of these) and not the consultant. The consultant’s role is to help these parties think more clearly about the ethical implications of their actions to optimize decision making” (2011, pp. 8–9).

Commenting on the example of a patient with decision-making capacity who wants to have life-sustaining treatment withdrawn, the document says:

The patient is the ethically appropriate decision maker…The consultant may guide discussion here in a way that enhances the decision-making authority of the patient, which is well-established by societal values and law (and presumably the institutional policy as well), and confirmed in the bioethics literature…Ethics consultants need to be sensitive to their personal moral values and should take care not to impose their own values on other parties. (2011, p. 9)

When there is intractable conflict among parties, the approach is procedural:

When agreement cannot be reached, the proper course of action can sometimes be determined by answering the question, “Who is the ethically appropriate decision maker?” Societal values often indicate who should be allowed to make the decision in the absence of agreement…[for example] a well-informed patient with decision-making capacity is generally allowed to accept or refuse any recommended treatment. (2011, p. 9)

Throughout ASBH’s discussion of the nature of ethics consultation, we see an appeal to established norms rooted in bioethical consensus as the standard of ethics. Based on the *Core Competencies*, it is apparent that ASBH adopts the Conventional Ethics model.

This interpretation of the *Core Competencies* is confirmed by some of its main architects. Mark Aulisio and Robert Arnold, two of the principal authors of the document, explain their conception of clinical ethics as follows:

Approximating moral truth…should not be the goal of ethics consultation as we conceive of it. There is a deep sense in which, on our view, “moral truth” is irrelevant for ethics consultation. How so? This is largely due to the fact that issues that arise in the clinic emerge in a context in which individuals retain their political rights to live according to their own moral views even if those views turn out to be false from some other particular moral point of view even, per hypothesis, the “correct moral view.”….We suggest that there is a deep sense in which clinical ethics consultation is more in the domain of the political than the moral, and more in the domain of practice than theory. Given its context, the appropriate question for ethics consultation is most often “Who should be allowed to decide?” rather than “Which view most approximates moral truth?” (2003, p. 279)

Elsewhere, Aulisio explicitly endorses a different sense of ethics for clinical ethics:

“Ethics” in clinical ethics consultation is importantly different from academic speculation in ethics. In the comfort of the classroom, one can adopt particular substantive moral views, consider their implications, and argue toward particular conclusions….“Ethics” for clinical ethics consultation, however, is profoundly different *even at this theoretical level*, precisely because of its *irreducibly contextual dimensions*. In clinical ethics consultation, issues are framed by certain social and political realities…such as fundamental societal values, law, and institutional policy, [which] also carry normative weight and so must inform decision making. Foremost among these is the social and political *fact* that all parties bring to the case certain moral and political rights grounded in our liberal constitutional democratic framework….[T]he focus, given this societal context, should be less on “who *is* right” than on “who *has* the right”…Thus, one of the most fundamental ethical questions for actual clinical ethics consultation is often “Who is (or are) the appropriate decision maker(s)?” or, to put it another way, “Whose values should be reflected in decision making?” (2003, p. 15)

Not only do these authors reject the idea that clinical ethics is concerned with moral truth, they go so far as to place it in the realm of politics rather than morality.

For Aulisio, clinical ethics is not about substantive moral questions of good and bad, right and wrong, or virtue and vice: “Moral truth per se, i.e., the substantively ‘correct moral view,’ has little to no role to play in [clinical ethics consultation]” (2019, p. 74). According to Aulisio, “no matter what any of the rest of us [including the ethicist] might think about the patient’s substantive values—whether or not he *is* “right” in some sense—he still *has* the moral and political *right* to make these decisions; his “having the right” stems from the societal value of autonomy as embedded in our liberal constitutional political structures” (2003, p. 16). Although he refers to some of the rights in question as “moral,” they should be understood as *political* or *legal* rights, because his view cannot affirm substantive moral rights without being internally inconsistent. The obligation to respect individuals’ values is a contingent social-political requirement, not a moral requirement. In Aulisio’s view, ethics consultation exists to serve the need of resolving value conflicts in health care, which is a pragmatic task. The goal is not to judge what is morally correct and do what is right, but to facilitate peaceful resolutions of value conflicts and “work toward a consensus that falls within the boundaries set by societal values, law, and institutional policy” (2003, p. 11). Aulisio provides an example of this approach in action:

imagine that an attending physician calls asks [sic.] for help in addressing a Jehovah Witness patient’s refusal of life-saving blood products. Irrespective of whether objective moral truth exists and is at odds with the patient’s Jehovah Witness beliefs, the focus of the consult would not (and should not) center on convincing the patient that his beliefs are “wrong” or “false” so that he is persuaded to agree with members of the care team, the ethics consultation team, or even members of his family (should they disagree with him). In fact, the moral truth or falsity of the views of any of the involved parties, patient’s included, is simply not relevant for addressing the issue. Irrespective of the existence of moral truth, patient, family members and caregivers alike have a right to be wrong! (2019, p. 74)

If the last sentence is meant to be a moral truth-claim, then Aulisio’s view is inconsistent. If not, then it must be referring to a non-moral right—a legal or political right. In that case, clinical ethics is not really about morality, but instead is a matter of law and politics.

In line with this view, Engelhardt maintains:

*Core Competencies* fails to recognize that the ethics of health care ethics consultants is not ethics in the usual sense of a morally canonical ethics. Its ethics is the ethics established at law and in enforceable health care public policy in a particular jurisdiction. Its normativity is a legal normativity, so that the wrongness of violating this ethics is simply the legal penalties involved and the likelihood of their being imposed….

the ethics of secular health care ethics consultation is not a normative ethics, as this might be understood apart from law and public policy…The ethics of secular health care ethics consultation is that ethics that has become dominant in the sense of becoming juridically canonical through being established at law and in embraceable public policy….

This “ethics” is a political or legal, not a moral fact of the matter. The canonical character and force of this ethics are juridical, not moral. (2011, pp. 129, 131, 141)

Jonathan Moreno agrees that clinical ethics has political normativity, not moral normativity. He argues that it is a procedural activity with roots in the political philosophy of classical liberalism:

the form ethics consultation has taken in our liberal society is rooted in political values. In the [*Core Competencies*] report’s general statement of the goal of health care ethics consultation, a critical phrase is the “resolution of ethical issues,” specified as the “resolution of conflicts.” Conflict resolution is not a chapter in any substantive moral philosophy, but it is a method for managing deep differences without recourse to violence. What defines the consultative situation as involving ethics is not that it is resolved by reference to a particular moral theory (this would be what the report calls an “authoritarian approach”) but that the controversial subject matter concerns conflicting *moral* values….the ethics consultation process is not a value-neutral one, but its values are mainly procedural, a view that emerges directly from classical liberalism. (2003, pp. 29–30)

The proceduralist approach to ethics is based on “contentless” processes that are devoid of “thick” metaphysical and moral content, with a focus on who should make the decision rather than what the decision should be (Bishop, 2018).

Edmund Howe, longtime editor of the *Journal of Clinical Ethics*, embraces the idea that clinical ethics is purely procedural. He claims that “ethical analysis—and, therefore, ethics consultants—can’t tell us which of two important and mutually exclusive values to choose; for example, it cannot tell us whether abortion is “right.”….Ethics consultants’ exceptional expertise is in knowing how ethical decisions should be made, not in which value or values should prevail” (2005, p. 2). In the same vein, the entry on clinical ethics in the *Encyclopedia of Bioethics* says that “at a general level, ethics facilitation as characterized by the ASBH is far more concerned with who has legitimate decision-making authority (i.e., who should be allowed to make a decision) than whether a single ‘right’ decision is made, and with building a consensus that respects that decision-making authority” (Aulisio and Younger, 2014, pp. 605–6). The proceduralism articulated by these authors is a standard feature of Conventional Ethics.

To sum up: We have seen two different ways of understanding the ethics of clinical ethics: Real Ethics consisting of objective moral norms grounded in moral truth, and Conventional Ethics consisting of conventional norms grounded in bioethical consensus. An examination of the theory and practice of clinical ethics suggests that it is best understood in terms of Conventional Ethics. The “elephant in the room” is that, on the reigning paradigm, “ethics” refers to a relative conventional standard rather than an objective moral standard.

## Critique of Conventional Ethics

This section is a critical evaluation of the Conventional Ethics paradigm. The first problem is that, like Real Ethics, it too faces the challenge of moral pluralism. Recall that the main objection to Real Ethics is moral diversity and intractable disagreement about moral truth and what is good, right, and virtuous. The main advantage of Conventional Ethics is supposed to be that it avoids this pitfall because it is procedural rather than substantive and it does not depend on partisan, content-full moral beliefs and values. Most of the authors cited above articulate the ideal of a value-neutral clinical ethics that is free of substantive moral content. Ethical conflict and uncertainty are meant to be resolvable using established norms and procedures that recognize the decision-making authority of autonomous patients and demand respect for their personal values.

The problem, however, is that the Conventional Ethics approach is not really value neutral, and it rests on substantive moral commitments that are not universally accepted. This includes a cluster of assumptions about autonomy, which tends to be equated with unconstrained self-determination and made an absolutely overriding consideration. One example is the claim that the personal moral beliefs, values, and preferences of patients must be respected and cannot be challenged or corrected on the grounds that they are false, unreasonable, or immoral. Another is the near-absolute right of patients to refuse any and all treatment, even when doing so could be considered morally wrong. These claims, as well as other common assumptions regarding medical decision making, depend on a substantive and contestable view of the nature, value, and weight of personal autonomy and preferences (Pellegrino & Thomasma, 1988; Bedford, 2011).^12^

Christopher Meyers, commenting on Aulisio and Arnold’s defense of ASBH’s ethics facilitation model, says:

On the surface, [their view] may sound like an endorsement of morally neutral tolerance, to the point of at least bordering on relativism. But I think that is an incorrect reading: Their view, again representative of much of the literature, has a deeply embedded and normative endorsement of the right to autonomy, as understood within a political environment, namely, “to live according to their own moral views, even if those views turn out to be false”…their emphasis on autonomy is also a clear endorsement of ethical truth, that is, to the truth of autonomy being a key, even *the* key, ethical principle. (2018, p. 58)

Evan DeRenzo takes a similar position to that of Aulisio and Arnold, according to which ethicists cannot provide the correct moral answers and should not make moral recommendations due to moral pluralism. She asserts:

We [clinical ethicists] are not—nor should we be—persons with moral answers. I am always startled when clinical bioethics consultants describe their role with phrases such as, “Bioethics consultants assist others in arriving at the most ethically justifiable solution.” My rejoinder is, “Most ethically justifiable to whom and on what grounds?” The faulty concept of ‘most ethically justifiable’ only demonstrates, to me, that the speaker confuses “moral expertise,” a reasonable term indicating a solid grounding in the full range of ethical perspectives coupled with the ability to engage others in substantive moral discussion, with the repugnant oxymoron “moral expert.” In a democratic society there is no such thing as a moral expert….

The concept of “the most ethically justifiable solution” is an insult to the citizens of a pluralistic society. Instead, in any given set of circumstances there will be a range of ethically permissible choices. Although the clinical bioethicist may be central in identifying that range, once the boundaries of acceptable choices have been set, the bioethicist’s role is to assist the primary decisionmakers, i.e., the patient, appropriately involved family and friends, and the health care team, arrive at a plan or solution that most closely approximates the patient’s wishes and that is not antithetical to a standard of best interest. (DeRenzo, 1994, pp. 384–5)

DeRenzo’s view is self-defeating. For she claims that if ethicists try to offer the correct, right, or “most ethically justifiable” answers, they run into the problem of moral pluralism, which she thinks is insuperable. At the same time, she believes that ethicists can offer answers about the “range of ethically permissible choices.” But the moral pluralism objection applies just as much to DeRenzo’s position. There is no difference in kind between the pluralistic challenge to the correct, right, or most ethically justifiable choices and to the range of ethically permissible choices. Whatever criteria are used to determine the range of ethically acceptable options will be non-neutral and content-full, not neutral and contentless. If “[t]he concept of ‘the most ethically justifiable solution’ is an insult to the citizens of a pluralistic society,” then the concept of the set of ethically permissible options—and, by implication, impermissible options—will be too. Moreover, DeRenzo’s claim that the goal of ethics consultation should be respecting the patient’s wishes and promoting the patient’s best interests is not neutral because it assumes a position on the nature and primacy of patient autonomy that not everyone shares, and it overlooks the fact that the question of best interests is highly disputed as well (Shea, 2020). Thus, to be consistent, DeRenzo should reject her own account for the same reasons she rejects the opposing one.

Another substantive moral commitment of Conventional Ethics is the assumption that conventional norms should have final and overriding authority with respect to resolving ethical conflicts. When there is a value conflict among different parties in a clinical encounter, and one party appeals to conventional norms while the other appeals to moral truths to support their respective positions, the conflict will be settled in favor of the former.

Take the case of a decisionally capable patient who is requesting that life-sustaining treatment be withdrawn against the recommendation of his physician and with the intention of bringing about his own death. According to some moral traditions (e.g., Catholic morality), the treatment in question is considered “ordinary” or morally obligatory, and it is morally wrong to intend one’s own death. The physician, who belongs to one of these traditions, believes that the patient’s decision is immoral and that she is morally obligated to protect the patient’s life and health and to avoid cooperating with his wrongdoing. The conventional norm is that capable patients have the right to refuse life-sustaining treatment for any virtually any reason, even if their decision is unreasonable or immoral, and even if they are intending their own death. On the Conventional Ethics approach, the “ethical” question will be “answered” and the conflict will be “resolved” by appealing to this rule and advising that the patient’s decision is “ethically permissible” and the “ethically required” course of action is to withdraw treatment. In this case, there could be a fundamental clash between what is morally right and what is required by conventional norms, especially regarding the ethical status of the patient’s choice, if not the physician’s obligation to respect it. Even if they align, the position that convention takes precedent over morality is a loaded and controversial stance rather than a neutral one.^13^

The second objection to Conventional Ethics is that it fails to address the real moral questions and issues at hand. Real Ethics is concerned with moral truth and with discovering and doing what is morally good, right, and virtuous. Conventional Ethics, by design, eschews moral truth in favor of socially constructed standards, which have at most only a contingent, accidental, and extrinsic relation to objective morality. It may be that conventional norms align with moral truths (whatever they happen to be). But the harmony is just a coincidence, because there is no necessary, essential, or intrinsic connection between them. When a clinical ethicist issues a recommendation about what ought to be done, it is not the case that a certain action or decision is “ethically appropriate/justified/permissible/obligatory” *because it is morally right*; instead, its ethical status is a function of conformity to established norms. Ethics recommendations track convention rather than morality.

Even after all the conventional norms are known, their application to the case is clear, and the verdict they entail is indisputable, there will still be a set of crucial questions left open, such as: Are the conventional norms *morally valid and binding*? Is the conclusion they entail *true*? Is the decision they support *morally justified*? In the day-to-day practice of clinical ethics, ethicists, health care professionals, patients, and family members usually are not permitted to engage in this kind of questioning and deliberation (without going outside the bounds of the practice). Most of all, they are not at liberty to dispense with the established ethical norms because they are deemed to be false or unjustified when considered from the moral point of view.

The problem with this approach is that questions about moral truth and justification are different from questions about conventional norms and how they should be interpreted and applied. They are also far more important—both theoretically and practically. Adams puts it well:

[Conventional Ethics is] premised on the belief that moral disagreement and confusion will always and straightforwardly turn on *what the conventional norms say*, not on *whether those norms are right (or justifiable)*. But there is no basis for this supposition.

Imagine people disagreeing, say, over whether sedation to unconsciousness is equivalent to euthanasia, whether allowing Grandpa to “pull the plug” on himself is letting him commit suicide, or whether a patient diagnosed with “total brain failure” is really dead. If the parties in such cases are in fact only disagreeing over how courts have ruled on these questions, what hospitals policies mandate, or what response the AMA *Code* gives, then of course sharing what those conventional sources say will resolve their problems—but such cases are surely rare. People in hospitals with serious moral concerns about what to do are not just quarreling over what the College of Physicians thinks is right or what institutional procedures allow; they disagree over—and want to know—what courses of action are *in fact right*, what decisions are *all things considered* ethically justifiable (or unsupportable). (2018, pp. 216–17)

According to the traditional understanding of morality found in the ordinary conception of ethics and in moral philosophy, morality is distinct from social convention, law, and politics.

In addition, the standard view affirmed by many philosophical and religious traditions is that morality has *overriding authority*: moral considerations trump other kinds of considerations when they come into conflict. As C.I. Lewis puts it, “In all the world and in all of life there is nothing more important to determine than what is right” (1955, p. 3). One application is that when it comes to determining how we ought to act, all-things-considered, morality takes precedent over individual belief, majority opinion, professional consensus, and law and public policy. The most famous spokesperson for the supreme authority of morality is Plato’s Socrates, who says that “each of us must neglect all other subjects and be most concerned to seek out and learn those that will enable him to distinguish the good life from the bad” (Plato, 1992, p. 289), and “he should look to this only in his actions: whether what he does is right or wrong, whether he is acting like a good or a bad man” (Plato, 2002, pp. 32 − 3). Indeed, it was this attitude that led Socrates to accept his unjust execution rather than commit an injustice himself.

For those who embrace the traditional understanding of morality as objective and overriding, this means that Conventional Ethics fails to consider the real moral questions and conflicts that arise in the clinic, because the conventional norms are not concerned with morality. That alone would be bad enough. To make matters worse, moral norms could be not just ignored but *subordinated* to conventional norms. In cases where morality and convention conflict, convention will win out, and the so-called “ethically appropriate” course of action will be to honor conventional norms while disregarding or violating moral norms. We have already seen how this happens in the previous case of the patient refusing life support, where Conventional Ethics says that the patient’s decision must be respected even if it is morally wrong, and ignores disputed moral questions about the obligation to conserve one’s life, the permissibility of intending death, the obligations of physicians toward their patients, and the permissibility of cooperation with evil.

Consider another example involving medical futility. Imagine that the family of an incapacitated patient is requesting to continue life-sustaining treatment that the physician believes is medically futile or inappropriate. To make it more concrete, imagine that the patient has suffered a neurological injury and is expected to remain permanently unconscious, and the hospital’s futility policy states that it is medically inappropriate to provide life-sustaining treatment to patients in such a neurologically devastated state. The attending physician, invoking the hospital’s policy, is attempting to place unilateral limits on the patient’s treatment and withdraw life support against the family’s wishes. The doctor believes that keeping the patient alive will not achieve a proper goal of medicine and will be non-beneficial because the patient’s quality of life is unacceptably low. The family believes that prolonging life, even without the possibility of conscious awareness, is a proper goal of medicine and will benefit the patient because life itself is an intrinsic good, the treatment is not disproportionately burdensome, and there is a duty to conserve life in this scenario.

On the Conventional Ethics approach, the “ethical” question will be “answered” and the conflict between the physician and the family will be “resolved” on the basis of the institution’s futility policy (and the law that permits the hospital to implement it). In this case, because the hospital’s policy stipulates that the treatment in question is medically futile according to its own futility criteria, the conventional norms will dictate that it is “ethically permissible” (and perhaps even obligatory) for the physician unilaterally to withdraw life support and let the patient die against the objection of the family. But this “resolution” could be morally incorrect. More to the present point, regardless of whether it is correct or incorrect, it doesn’t in any way address the real moral questions at stake or the deeper moral issues underlying the disagreement between the two parties, including the proper goals of medicine, the nature of benefit and harm, the value of human life, the duty to conserve life, the value of conscious awareness, and whether unilateral physician decision making is morally justified. The conflict will be “settled” according to the established norms, but the more fundamental moral questions and debates will not even be addressed, let alone resolved. In fact, there might not be anything genuinely *moral* in the ethics process at all. The real moral issues will be skirted and convention will replace morality.

For those who believe that morality takes priority over convention and that our central task as moral agents is to try to discover the truth, pursue the good, be virtuous, and do what is right, this will be a surprising and strange feature of Conventional Ethics and one that diminishes the value of clinical ethics so understood. (And it is another occasion where ethicists might experience a form of Anscombian jaw-misalignment when talking about what is “ethical.”)

The third problem with Conventional Ethics is that, on this approach, clinical ethics fails to meet some important needs and expectations of the people it is meant to serve. Sometimes health care providers, patients, and families call upon ethicists because they want to know the conventional norms governing health care or the legal and institutional processes governing their practice, or they encounter a difficult practical problem or interpersonal conflict that can benefit from the mediation and facilitation skills of ethicists. But sometimes people seek ethics consultation and education because they are looking for help in determining what is good, right, and virtuous—full stop. They want to know what the morally correct answer or decision is because they want to do the right thing, regardless of what the conventional norms might say. In other words, what they really seek and need is Real Ethics. But, on the Conventional Ethics paradigm, Real Ethics is something ethicists are not supposed to offer. Some proponents of the view go so far as to say that ethicists would be acting unprofessionally and inappropriately if they attempted to engage in Real Ethics.^14^

Because morality is one of the highest—if not the very highest—priorities in the life of a morally committed person, the inability of clinical ethics to speak to questions of moral truth—to assist those seeking ethics guidance with determining what is truly good, right, and virtuous, and not just what is conventionally accepted—is a major weakness of the practice, especially a practice devoted to “ethics.” The most important thing from the moral point of view is morality, and Conventional Ethics cannot address this all-important subject.

On top of being unhelpful, Conventional Ethics can also be confusing and misleading to those looking for ethics consultation and education, because people often assume that clinical ethics is about Real Ethics when that is not in fact the case. Engelhardt writes:

the use of ‘ethics’ in clinical ethicists and ethics consultation does not have the moral meaning that most persons associate with the term….Without renaming the practitioners as well as their consultations, and in addition making provision for access to alternative ethicists, who may be real moralists, there is a conflict between the interest in professional recognition and the interest adequately to inform patients, families, and others of the moral expertise offered by clinical ethics consultation. Patients, families, and others may receive a consultation they did not want and not have access to the moral advice they had sought. (2009, pp. 303–4)

Conventional Ethics leads to false expectations on the part of patients, families, health care professionals, and institutions, because some people seek ethics advice expecting Real Ethics but get something very different.

In my view, the preceding three objections, when taken together, amount to a strong argument against the Conventional Ethics paradigm of clinical ethics.

## Recommendations

I conclude by offering some brief suggestions for how clinical ethics can alleviate some of the problems raised in the previous section.

First, there should be more clarity about what clinical ethics is and is not, in both the scholarly literature and clinical practice. For instance, in contexts where Conventional Ethics has been universally adopted, it may be wise to rename the field so that it no longer includes the term ‘ethics.’ That would prevent clinicians, patients, families, and institutions from misunderstanding the nature of clinical ethics and the role of the ethicist because it would be clearer that they do not correspond to the ordinary notion of ethics or Real Ethics. Perhaps a more fitting name would be “values counselor” or “conflict mediator.” (I leave it to others to come up with something better.)

Second, ethicists and institutions should be more transparent about the kind of ethics behind their practice, whether it be Real Ethics, Conventional Ethics, or something else. They should also disclose the normative framework governing their practice, since, as we have seen, neither Real Ethics nor Conventional Ethics can succeed in being value neutral. That way, clinicians, patients, and families would be more aware of the conception of ethics and the substantive moral values that inform the ethicist’s recommendations and the institution’s ethics process. By increasing transparency, those who are served by ethicists will be better informed about the ethical dimension of the health care they provide or receive, and their moral agency will be enhanced because they will be able to recognize how the ethical norms operative in the ethics process relate to their own moral commitments.

To return to the futility case from earlier, if the family is aware that the ethical principles supporting the institution’s futility policy are conventional standards rather than moral truths, they will be more likely to avoid mistakenly assuming that the physician’s conflicting judgment necessarily has some kind of objective moral authority, and they may be more inclined to challenge it by shifting the conversation to the domain of Real Ethics, where their moral beliefs and values will not automatically be overridden by the conventional norms. This would be a more open, fair, and authentically moral process of engagement between disagreeing parties.

Finally, in light of the problems with Conventional Ethics, there are good reasons to adopt a model of clinical ethics that involves Real Ethics. This does not mean that ethicists should use the “authoritarian” model of ethics consultation by acting as the primary decision maker and imposing their own view on all other parties, although in some cases there may be a need for them to speak the moral truth as they see it, in a spirit of persuasion rather than coercion. Instead, it means that ethicists, in their capacity as advisors and educators, should engage with questions of morality as well as convention. There are multiple ways to implement the Real Ethics approach, and they will not be explored here. My primary goal in this article is to critique Conventional Ethics, not to defend a specific version of Real Ethics, which would require a longer treatment. Whatever form it takes, clinical ethics should address moral issues, conflicts, and disputes in some way.

Clinical ethicists provide a vital, valuable, and noble service. Ultimately, if they are to better understand the nature of their profession and meet the needs of the people they serve—especially the need for *moral* advice, guidance, and education—they must move beyond the vague and ill-defined common notion of ethics and embrace one that is clearer, richer, and more real. That way, their “teeth can come together in a proper bite” when they talk about ethics.

## Footnotes

1 The discussion that follows is meant to cover all three of the major roles and activities of clinical ethicists, but the primary focus will be ethics consultation.

2 My focus is secular clinical ethics in the United States, leaving aside clinical ethics as it is understood and practiced in religious contexts (e.g., Catholic health systems) and in other countries.

3 There is no consensus on the exact definition of morality, but there are some characteristics that are often said to define it. Putting them together, we can say that morality refers to codes of conduct and character that are normative, practical, rational, universalizable, impartial, social, public, overriding, applicable to all moral agents/persons, and related to human good/well-being (Pojman, 2016, ch. 1; Gert & Gert, 2020).

4 The notion of objective moral truth used in this article is meant to be generic and consistent with a variety of metaethical theories, such as ethical naturalism, non-naturalism, supernaturalism, and some versions of constructivism and contractualism. Contrasting views are *moral nihilism*—valid moral norms do not exist—and *moral relativism*—the validity of moral norms depends on individual opinion or social convention.

5 Real Ethics is a *metaethical* view that does not entail any specific position in normative ethics and is compatible with a variety of approaches, including traditional ethical theories such as utilitarianism, deontology, and virtue ethics; mid-level theories such as principlism; anti-theories such as casuistry; and theories that ground medical ethics in the nature and ends of medicine.

6 In their taxonomy of the various positions in the debate over ethics expertise, Abram Brummett and Erica Salter identify “moral realism” (basically the same thing as what I call “ethical objectivism”) as one of the metaethical views found in the scholarly literature: “The moral realist approach to justification claims that recommendations are better or worse by how well they correspond to some context-independent notion of moral truth” (2019, p. 57).

7 A possible exception to this rule is cases where there is no established norm that covers the ethical issue in question, and conventional norms are not sufficient for an ethics recommendation. In these cases, which are rare, it might be held that ethicists are permitted to engage in Real Ethics to cover uncharted and unsettled territory.

8 Here is a more technical way to explain it. According to Conventional Ethics, the truth-maker/justifier for ethical propositions (like the ten listed above) is not objective morality; rather, it is conventional norms. It may be the case that ethical propositions are true/justified according to both morality and convention, but the overlap will be contingent rather than necessary. The two standards of ethical truth/justification are distinct and possibly inconsistent. Moreover, the conventional norms do not have any essential and intrinsic relation to moral truth/justification and are not guaranteed to track it. It is not the case, for example, that ethical propositions are made true/justified by their correspondence to conventional norms, and conventional norms are made true/justified by their correspondence to objective moral reality or moral facts.

9 This is not to say that everyone who favors Conventional Ethics is an ethical relativist. Many (perhaps most) clinical ethicists reject relativism as a metaethical theory. What I mean is that on the Conventional Ethics approach, the *operative normative standard* for clinical ethics is a relativistic one, regardless of whether its proponents endorse relativism as a metaethical theory. That is why I refer to it as “de facto” ethical relativism.

10 Proponents of Real Ethics and Conventional Ethics can agree that consensus is a proper *epistemological method* to use in clinical ethics, but they will disagree about what the relevant *normative standard* is. For example, one could hold that the set of conventional norms rooted in bioethical consensus is not the normative standard that determines what is ethical; instead, it is an epistemological standard that gives us a means of knowing or forming justified beliefs about what is objectively morally correct. This view falls under Real Ethics because it takes the relevant normative standard to be morality rather than convention. And, it is worth noting, this approach may be the best way to alleviate the epistemological worry that Real Ethics lacks a reliable method for figuring out the moral truth and resolving moral disagreements in a pluralistic society. There is a closely related view that holds that bioethical consensus is the proper epistemological method for knowing what is ethical, objective moral truth is the *ultimate* normative standard that determines what is ethical, and the set of conventional norms is the *proximate* and *operative* normative standard for clinical ethics. This approach falls under Conventional Ethics because the ethical standard used in clinical ethics is conventional norms rather than the objective moral law. Relatedly, proponents of Real Ethics can recognize that conventional norms have moral relevance and factor into the determination of what is morally correct; but it will be in virtue of their relation to morality, which is the operative normative standard.

11 “Regarding *value*, we realize that there are values embedded in many different domains (e.g., law, morals, professional practices, various communities, individual conceptions of the good). We use *value* as a general term to capture the various normative dimensions of issues that emerge in health care. Value uncertainty or conflict often arises because of competing values from these different domains” (2011, p. 2, n. 2). Saying that ethics deals with values and normativity is too broad of a definition because it overlooks the fact that there are multiple kinds of value and normativity, not just the moral kind.

12 Some influential moral systems (e.g., Kantian deontology, natural law theory) maintain that the value of autonomous choices and personal preferences, and the obligation to respect them, at least sometimes depend on whether they accord with the objective moral law. More generally, Real Ethics is not neutral or relativistic about individual values: it subjects them to normative evaluation and says what our values *ought* to be. Moral norms apply to everyone regardless of their personal beliefs and preferences.

13 For a longer argument that the conventional and proceduralist ethical framework that is dominant in secular clinical ethics is not in fact neutral and contentless but instead involves partisan and content-full moral and metaphysical commitments, see Brummett and Eberl, 2022.

14 This is the apparent position taken by ASBH. Its *Code of Ethics and Professional Responsibilities for Healthcare Ethics Consultants* states: “Consultants should preserve professional integrity by not engaging in activities that involve giving an ethical justification or stamp of approval to practices they believe are inconsistent with agreed-upon ethical standards. If a conflict involving the consultant’s personal core beliefs or values arises in the course of performing [ethics consultation], the consultant should recuse himself or herself from the case after securing the services of a replacement. For example, [ethics] consultants who have a strong moral objection to artificial reproductive technologies (ARTs) should recuse themselves in consultations involving ARTs and should not agree to provide [ethics consultation] in a setting where issues related to ARTs routinely arise. If no replacement is available, the primary obligation of the [ethics] consultant is to maintain professional integrity” (2014, p. 2).
